# Stability of Amines with Minerals and Metal Salts in Hydrothermal Fluids

**DOI:** 10.1021/acsomega.6c05119

**Published:** 2026-07-20

**Authors:** Yiju Liao, Alexandria Aspin, Ziming Yang

**Affiliations:** Department of Chemistry, 6918 Oakland University, Rochester 48309, Michigan, United States

## Abstract

Amines are key intermediates in biogeochemical nitrogen cycling and are potential precursors to amino acids and other biomolecules in hydrothermal systems on Earth and ocean worlds. While their reactivity and chemistry under high-temperature conditions have been previously studied, far less is known about how inorganic components such as metal ions and minerals influence amine stability and transformation pathways. Here, we investigate the hydrothermal reactivity of three representative amines (benzylamine, cyclohexylamine, and cyclohexylmethylamine) at 250 °C in the presence of Al-, Fe-, Mg-, and Zn- bearing minerals and metal salts. The amines exhibited moderate conversion and formed products including alcohols, alkenes, aldehydes, and diamines. Minerals and dissolved metal salts produced only minor enhancements or suppressions in total amine conversion and did not substantially alter product distributions or reaction pathways. These results indicate that small aliphatic and aromatic amines are not strongly affected by metal- and mineral-rich hydrothermal fluids. The limited influence of inorganic materials suggests that amines could persist and accumulate in hydrothermal environments, supporting their role as chemical precursors in prebiotic synthesis.

## Graphical abstract

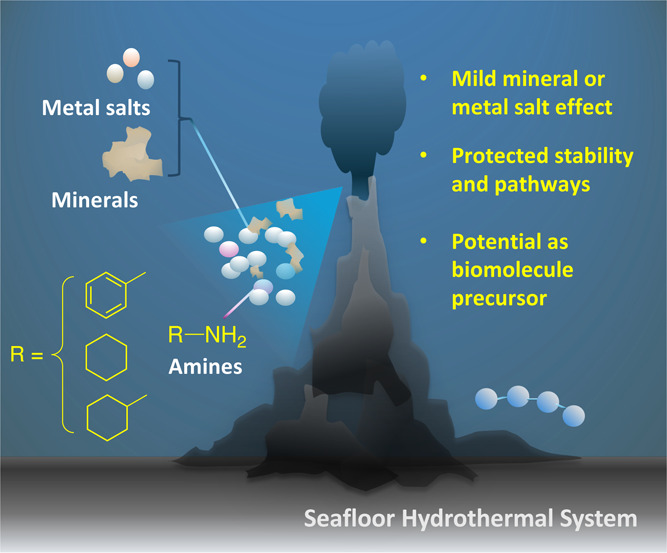

## 1 Introduction

Hydrothermal environments, which are located deep within Earth’s oceanic crust, are of great interest due to their ability to host diverse ecosystems despite the high temperatures and pressures characteristic of vent environments.^1,2^ Life in these systems rely on the hydrothermal fluids for energy and nutrients, which are often circulated through Earth’s crust and the surrounding seawater.^3,4^ Similar systems are also believed to exist beyond Earth, such as in Saturn’s moon, Enceladus.^5–7^ Studying these environments not only deepens our understanding of Earth’s subsurface biosphere but also informs the ongoing search for life beyond Earth.

Several theories suggest that hydrothermal processes may have played a role in prebiotic organic synthesis and possibly contributed to the origin of life on early Earth.^8–10^ Organic transformations of various functional groups have been investigated under hydrothermal conditions to determine their reactivity, stability, and reaction mechanisms.^11,12^ Specific pathways and interconversions among compounds such as alkanes, alkenes, alcohols, ketones, and carboxylic acids have been discovered to be prevalent under hydrothermal conditions and studied in laboratory settings.^13–15^ However, natural hydrothermal systems also contain inorganic materials such as dissolved gases or metals, as well as minerals, which may influence organic transformations. For example, iron sulfide minerals such as pyrrhotite increased ketone reactivity under hydrothermal conditions and altered the reaction pathways by favoring the reduction of ketones to alcohols and alkanes.^16^ Similarly, the selective reduction of alkanes to alkenes occurred with iron and nickel,^17^ and the oxidative decarboxylation of organic acids was facilitated by dissolved copper salts.^18^ Recent research also revealed the oxidative role of copper­(II) and iron­(III) in hydrothermal reactions of alcohols, aldehydes, and alkenes.^19–21^ Hydrothermal interactions among organics with minerals or metals thus not only represent the geochemical processes in natural hydrothermal environments but also provide new avenues for using “geomimicry” in sustainability and green chemistry.^22,23^

Beyond the extensively studied hydrothermal transformation of organic carbon, nitrogen cycling under extreme conditions also governs the fate and transport of biomolecules such as amines, amino acids, and amides.^24–26^ Organic nitrogen plays important roles in metabolic cycles in the subsurface biosphere, and in the synthesis of biomolecules in hydrothermal systems on Earth and possibly on other planets.^8,27,28^ Organic nitrogen compounds, such as amines, are not only the key components of amino acids and proteins forming the backbone of organisms, but they are also precursors to amino acids and peptides in a prebiotic context. In natural hydrothermal systems, amines undergo various transformation pathways. For example, deamination of amines can result in the formation of alcohols, alkenes, and imines,^24,29^ while amines can also act as ligands binding to metal ions, potentially influencing the mobility and speciation of metals and minerals in hydrothermal fluids.^30^ Thus, investigating the reactivity and stability of amines under geologically relevant hydrothermal conditions could help to better understand the geochemistry of organic nitrogen in the deep ocean, energy flow, and metabolic cycles in those extreme environments.

Hydrothermal transformation pathways of amines can vary depending on the specific environmental conditions. Previous studies have identified the primary deamination pathways of amines, which involves formation and substitution of aminium ions, followed by oxidation to aldehydes and/or further transformations to form imines or secondary amines (Figure 1).^24,31^ In certain cases, particularly when the amine group is bonded to a secondary carbon, alkene formation can also occur, either through dehydration of the intermediate alcohol or via direct elimination of the amine group.^31,32^ These amine pathways are also shown to be subject to temperature, pH, fluid composition, as well as the structure and chemical properties of amines. However, few studies have investigated how the presence of minerals or dissolved metal ions influences amine stability or reaction pathways in hydrothermal fluids, which limits the understanding of amine compounds in natural and complex hydrothermal systems.

**Figure 1:**
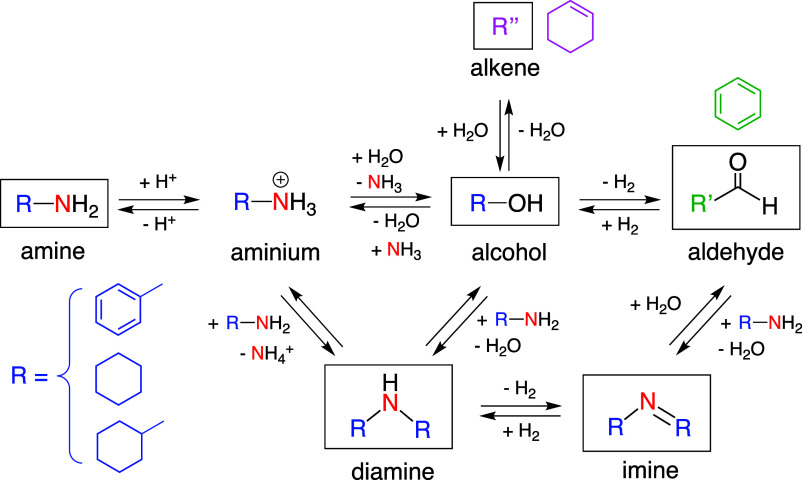
Proposed reaction pathways for the amines (e.g., benzylamine, cyclohexylamine, and cyclohexylmethylamine), based on the observed product distribution, under the hydrothermal conditions.

In this study, we investigated the effects of minerals and their corresponding metal ions on the reactivity and pathways of structurally diverse amines through laboratory-simulated hydrothermal experiments. Three model amine compounds (i.e., benzylamine, cyclohexylamine, cyclohexylmethylamine) including both aliphatic and aromatic structures were selected, which reactivity and pathways under metal/mineral-free hydrothermal conditions have been recently examined.^31^ Also note that the selected amines are all primary amines, which may not represent a full spectrum of amine structures (e.g., secondary or tertiary amines) in oceanic hydrothermal systems. Although the present study could be limited in the substrate scope, the identified amine hydrothermal chemistry and reaction pathways would be useful in predicting and interpreting other amine-containing compounds (e.g., amino acids) under hydrothermally or prebiotically relevant conditions. Gibbsite (Al­(OH)_3_), brucite (Mg­(OH)_2_), corundum (Al_2_O_3_), hematite (Fe_2_O_3_), magnetite (Fe_3_O_4_), sphalerite (ZnS), and pyrrhotite (FeS) were chosen for this study because they either are found near hydrothermal vents or have been shown to play a role in surface catalysis or adsorption in organic hydrothermal transformations.^16,33^ Compared to sulfide and iron oxide minerals that are commonly observed in deep-ocean hydrothermal vents, gibbsite and corundum are naturally formed on land through weathering and other geological processes, which may represent minerals in terrestrial hydrothermal systems. Chloride salts were selected because chloride is highly abundant in oceanic environments, and the metal cations in these salts (Fe^3+^, Al^3+^, Mg^2+^, and Zn^2+^) correspond to the same metals present in the minerals included in this study. CuCl_2_ was also included because Cu­(II) is widely distributed at the seafloor from the hydrothermal dissolution of copper-bearing minerals, such as chalcopyrite,^34,35^ but also due to its strong and selective effects on organic functional interconversions.^18–20^ The selection of NaCl is due to its abundance in seawater and the potential effect on hydrothermal stability of amino acids.^32,36^

## 2 Experimental Section

### 2.1 Materials

Organic compounds including benzylamine (99.5%), benzaldehyde (99.5%), cyclohexylamine (99.9%), dibenzylamine (99%), cyclohexanol (98%), benzyl alcohol (99.5%), cyclohexane (99.9%), cyclohexanemethanol (98%), bis­(cyclohexanemethyl)­amine (99%), dicyclohexylamine (98%), and cyclohexene (98%) were purchased from Sigma-Aldrich. Minerals including Al_2_O_3_ (≥98%), Al­(OH)_3_ (≥97%), Fe_2_O_3_ (≥98%), Fe_3_O_4_ (≥98%), FeS (≥98%), Mg­(OH)_2_ (≥98%) and ZnS (≥97%) were purchased from Sigma-Aldrich and rinsed with deionized water and dried before use. Chloride salts, including CuCl_2_ (≥99%), NaCl (≥99%), FeCl_3_ (≥98%), AlCl_3_ (≥98%), MgCl_2_ (≥99%), and ZnCl_2_ (≥99%) were also obtained from Sigma-Aldrich and used as received. Dichloromethane (DCM, 99.9%) was obtained from VWR and used as the solvent for the organic extraction. Decane (99%) was purchased from Sigma-Aldrich and used as the internal standard for the gas chromatography (GC) analysis. Deionized water (18.2 MΩ·cm) was obtained from a Barnstead Nanopure system. The phosphate buffers were prepared using phosphoric acid (85.0 wt. % in H_2_O) and monosodium dihydrogen phosphate (≥99.9%), which were purchased from Sigma-Aldrich.

### 2.2 Methods

The hydrothermal experiments were conducted following previously developed methods.^31,37^ Briefly, 0.1 molal of amine (e.g., benzylamine, cyclohexylamine, or cyclohexylmethylamine) in 0.3 mL Ar-purged deionized (DI) water was loaded into fused silica glass tubes (2 mm × 6 mm, GM Associates). In mineral experiments, two equivalents of mineral powder (0.2 molal) were added to the quartz tubes, followed by the addition of 0.3 mL Ar-purged phosphate buffer solution (PBS; pH 2.2; 50 mM). The lower pH was chosen to facilitate the formation of aminium ions (protonated amines), allowing a higher rate of deamination than that at higher pH.^31^ The mineral surface area was measured using the Brunauer–Emmett–Teller method with N_2_ adsorption in a Micromeritics ASAP 2020.^100^ In metal salt experiments, 0.3 mL of 0.1 molal Ar-purged metal salt solutions (buffered to pH 2.2) was added to the reaction tubes. The buffer pH of 2.2 was selected to ensure the amines (e.g., benzylamine) were mostly protonated to promote the deamination.^24^ The pH of each starting solution with dissolved salts was measured at room temperature and was in the range of 3–4. Buffer catalysis was previously determined to be negligible by comparing reaction kinetics after doubling the buffer concentrations.^38^ Buffers were prepared according to calculations performed using the revised Helgeson–Kirkham–Flowers (HKF) equations of state and associated thermodynamic data,^39,40^ calculated with the software EQ 3/6 and SUPCRT92.^41,42^

The oxygen in the silica tubes was removed through three freeze–pump–thaw cycles before sealing using an oxyhydrogen torch under vacuum. The tubes were then put inside a preheated GC oven at 250 °C for up to 72 h. The experimental temperature was chosen based on the relative reactivity of amines studied in the recent work.^31^ The experimental pressure was at water saturation pressure (*P*_sat_ ∼ 40 bar at 250 °C). Although the reaction pressure may not represent higher pressure conditions in natural deep-sea hydrothermal fields, previous studies have shown that pressure did not have a substantial effect on certain organic transformations (e.g., ketones) under similar hydrothermal conditions.^14,15^ However, it is still likely that the reaction rates of amines could change at higher pressures, since the present study did not specifically examine the pressure effect. The hydrothermal reactions were quenched by submerging the silica tubes in a cold-water bath. The samples were extracted for analysis by the methods previously developed.^31,43^ In brief, 3.0 mL of DCM containing 8.8 mM decane was used to extract the organic products from the mixture through sonication. Subsequently, the solutions were alkalized by adding 60 μL of 5.0 M NaOH to neutralize any protonated amines and ensure complete collection in the organic phase. A second set of extractions was conducted without the addition of NaOH to determine whether organic acids were produced. The organic products were analyzed and quantified on an Agilent 7820A GC equipped with a poly capillary column (5% diphenyl/95% dimethylsiloxane) and flame-ionization detector. The GC oven was programmed to start at 50 °C (hold 8 min), ramp at 10 °C min^–1^ to 220 °C (hold 10 min), and ramp at 20 °C min^–1^ to 300 °C (hold 5 min). The injector temperature was set at 300 °C. Gas chromatography–mass spectroscopy (Agilent 7890*A*/5975C) was also used to confirm the product identification based on ion fragmentation patterns in the mass spectra, along with retention time matching with analytical standards. Product quantification was achieved via five-point linear calibration curves (*R* ^2^ ≥ 0.995) that produced response factors relative to the fixed internal standard, decane. Each hydrothermal experiment was conducted in duplicate, and the reported data represent the average with error bars showing one standard deviation.

## 3 Results and Discussion

### 3.1 Effect of Minerals on Amine Reactivity

The effect of minerals on the product distribution of benzylamine, cyclohexylamine, and cyclohexylmethylamine were examined at 250 °C after 24 or 72 h, depending on the amine reactivity (Figure 2). Shorter reaction time (24 h) was chosen for benzylamine because of its higher reactivity compared to cyclohexylamine and cyclohexylmethylamine, while maintaining a decent and comparable amine conversion between 25% and 45%. Among the studied amines, benzylamine was the most reactive, with ∼31% conversion after 24 h (Figure 2a). The major products include dibenzylamine (∼20 mol %), accompanied by appreciable amounts of benzyl alcohol (∼10 mol %) and only minimal amounts of benzaldehyde. The formation pathways of benzylamine products are depicted in Figure 1, which have been identified under similar hydrothermal conditions in a recent work.^31^ In the presence of minerals, the overall conversion of benzylamine and the product distribution were not much affected. Gibbsite, magnetite, pyrrhotite, and brucite slightly enhanced its reactivity (34–36% conversion), generally increasing dibenzylamine formation while reducing benzyl alcohol production. In contrast, corundum, hematite, and sphalerite slightly inhibited benzylamine conversion, lowering down to ∼26% conversion by mainly decreasing the benzyl alcohol formation.

**Figure 2:**
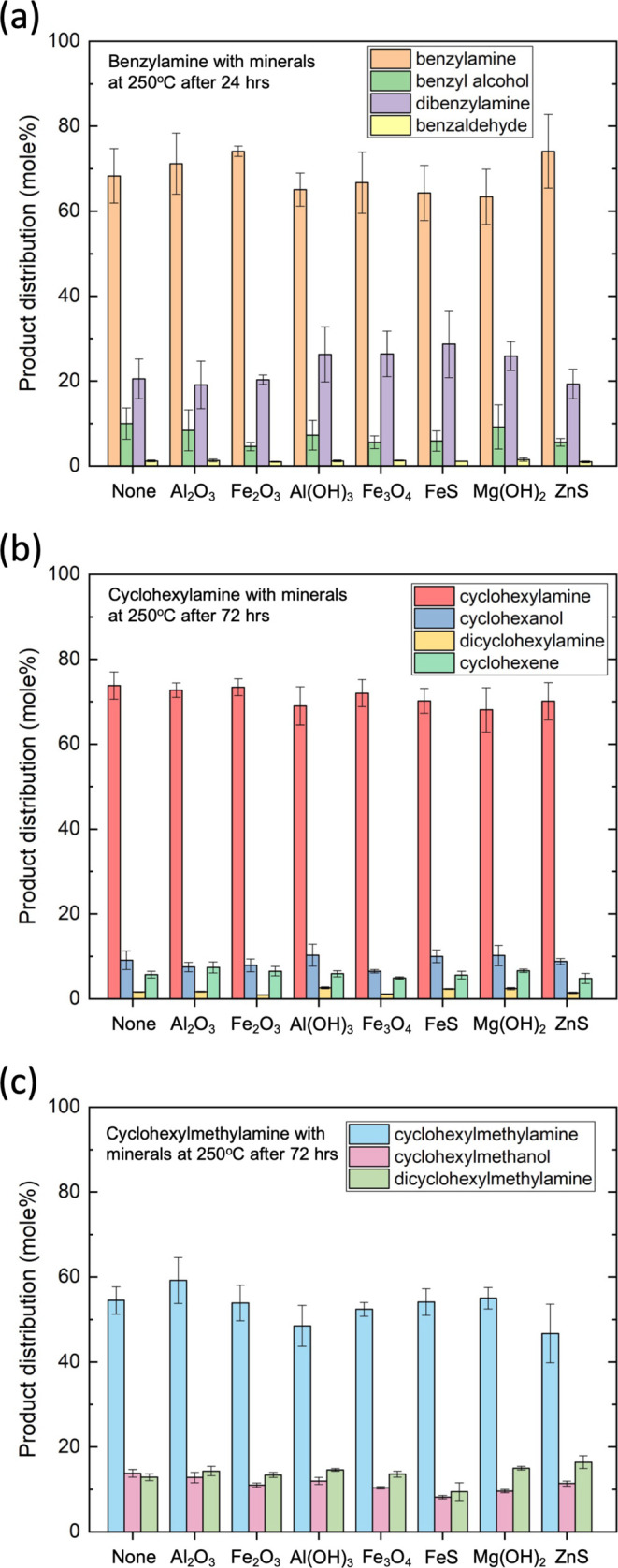
Product distribution of hydrothermal transformations of benzylamine (a), cyclohexylamine (b), and cyclohexylmethylamine (c) in the absence and presence of minerals at 250 °C after 24 or 72 h. Error bars represent one standard deviation of duplicate experiments.

Cyclohexylamine reacted the slowest, with ∼26% conversion after 72 h in only water (Figure 2b). Major products include cyclohexanol and cyclohexene, accounting for ∼9 mol % and 6 mol %, respectively. Dicyclohexylamine was also formed; however, it represented <2 mol % of the products formed. In the presence of gibbsite and brucite, the conversion of cyclohexylamine increased slightly to 31–32%, while the other minerals showed even less of an effect on cyclohexylamine’s conversion and product distribution. Cyclohexylmethylamine reacted faster than cyclohexylamine, with ∼45% conversion after 72 h (Figure 2c). Cyclohexylmethanol and dicyclohexylmethylamine were the only two products observed, and were comparable in concentration, both at roughly 13–14 mol %. The presence of minerals also had a little effect on the overall conversion of cyclohexylmethylamine, with gibbsite, magnetite, and sphalerite increasing the conversion to ∼47–53%, and corundum decreasing the conversion by roughly 4%. In general, the presence of minerals increased the formation of dicyclohexylmethylamine while limiting the formation of cyclohexylmethanol, however, this effect is minimal.

These observations indicate that the minerals studied exert only modest effects on amine conversion and product distribution. Although a fixed molal ratio between mineral and amine (2:1) rather than a fixed total mineral surface area was used in these mineral experiments, the difference between mineral surface areas (in a range of 5–12 m^2^/g) was only within a factor of two or three, and any strong catalytic effect should not be muted by the lesser surface area of minerals used in the present study. The small changes suggest that surface interactions or other mineral-mediated effects may play a limited, subtle role in controlling reactivity. Potential mechanisms for these minor effects, such as surface adsorption, interfacial water structuring, and mineral dissolution, are further discussed below (Section 3.3).

### 3.2 Influence of Inorganic Salts on Amine Reactivity

The metal salt solutions also showed a nonsubstantial effect on amine conversion under the studied conditions (Figure 3). To maximally reveal any metal salt effect on amines and minimize secondary reactions, shorter reaction times (4 or 24 h) with lower amine conversion (e.g., 10–20%) were chosen to study. In benzylamine experiments, most tested salts mildly increased the amine conversion from 15% to 22–30% after 4 h, except for NaCl, which showed no measurable effect (Figure 3a). The product distributions were also not much affected, in which the metals selectively increased the formation of benzyl alcohol from benzylamine, suggesting the deamination may be promoted in the presence of metal salts. This contrasts with a previous study, which reports that similar salts (e.g., AlCl_3_ and FeCl_3_) inhibited the deamination of 2-phenylethylamine, while CuCl_2_ had little effect on the overall amine conversion.^32^ In that study, the metal salts collectively suppressed the elimination deamination pathway leading to alkene formation, while promoting the substitution deamination to the corresponding alcohol. Here, benzylamine is structurally restricted to deamination via the elimination pathway, which may explain the enhanced conversion observed in the presence of metal salts. Additionally, CuCl_2_ facilitated the oxidation pathway to benzaldehyde, which is consistent with its oxidizing effect reported in previous studies.^43^

**Figure 3:**
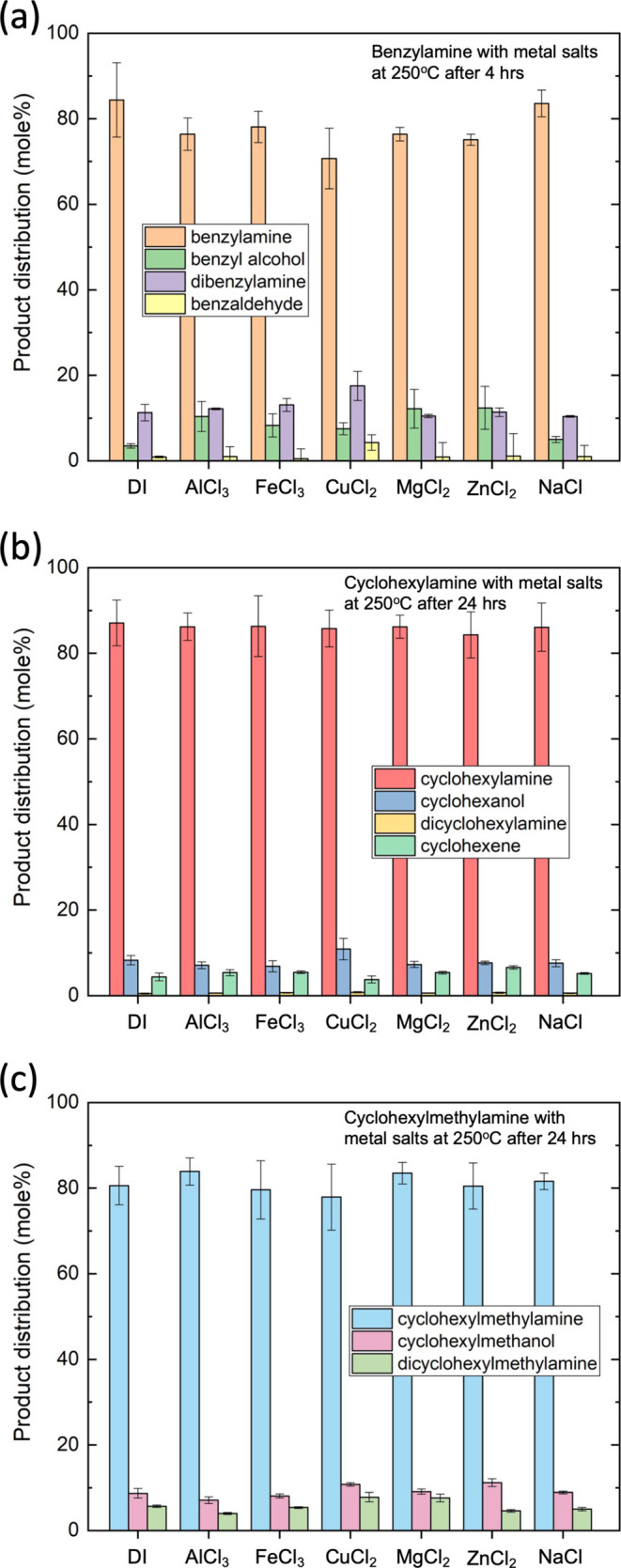
Product distribution of hydrothermal transformations of benzylamine (a), cyclohexylamine (b), and cyclohexylmethylamine (c) in the absence and presence of metal salts at 250 °C after 4 or 24 h. Error bars represent one standard deviation of duplicate experiments.

In comparison, the metal salts had a smaller effect on the reactivity of cyclohexylamine and cyclohexylmethylamine than that of benzylamine (Figure 3b,c). Cyclohexylamine conversion increased slightly from 13% up to 16% in the presence of metal salts after 24 h, with minimal changes in product distributions. Cyclohexanol and cyclohexene remained the dominant products, while dicyclohexylamine was a minor product. Cyclohexylmethylamine conversion ranged from 16 to 22% in the presence of metal salts, comparable to that in pure water (∼20%). Product distributions also changed to a small extent, with cyclohexylmethanol and dicyclohexylmethylamine continuing to be the major products. These findings suggest that simple amine structures are relatively stable in metal- and mineral-rich hydrothermal environments, allowing for accumulation in prebiotically relevant settings. This stability may also have implications for the origin of life on Earth and other ocean worlds with hydrothermal activity, as amines serve as key precursors to more complex biomolecules such as amino acids and peptides.

### 3.3 Stability of Amines in Oceanic Hydrothermal Systems

The experimental results demonstrate that the three simple amines, benzylamine, cyclohexylamine, and cyclohexylmethylamine, largely retain their stability under hydrothermal conditions, despite exposure to metal ions/mineral-rich environments. Such stability mirrors observations for amino acids in a recent study,^32^ in which decomposition pathways such as decarboxylation and deamination are strongly suppressed by the presence of metal cations and high ionic strength. In both systems, stabilization of organic nitrogen likely arises from a combination of protonation, weak complexation, and solvent-mediated effects that inhibit the C–N bond cleavage or formation. Under acidic conditions, protonation reduces amine nucleophilicity and can impede pathways requiring nucleophilic attack,^38,44^ while the high ionic strength of chloride-bearing fluids and the coordination of Fe^3+^, Cu^2+^, and Al^3+^ with water and organic ligands could also reduce the reactivity of organic nitrogen in salty hydrothermal solutions.^32^

Although minerals did not substantially alter product distributions in this study, they may still influence the fate of amines through adsorption or interfacial reactions that can introduce mild, mineral-dependent kinetic effects without fundamentally changing reaction pathways. Under acidic conditions, protonated amine groups (–NH_3_^+^) can electrostatically adsorb onto negatively charged oxide or hydroxide surfaces, forming outer–sphere complexes that may limit amine dissolution and other reactive aqueous pathways.^45^ Computational studies suggest that adsorbed molecules can be coupled to mineral surfaces through electrostatic interactions and water bridges, maintaining hydration shells that may limit direct electron transfer and suppress catalytic bond cleavage.^46^ The structuring of interfacial water induced by mineral hydration shells could also modify local activities of reactive hydroxide species,^47^ potentially inhibiting amine reactions. While mineral adsorption mechanisms such as noncovalent or electrostatic adsorption may contribute to the stability of organic matter in mineral-rich environments,^48^ the modest mineral effect observed in the present study suggests that stabilization by mineral surfaces may not substantially affect amine transformations under hydrothermal conditions.

In addition, mineral dissolution can also occur in hydrothermal fluids. Previous studies have shown the dissolution of hydroxide minerals such as gibbsite and brucite during laboratory hydrothermal experiments, including detailed examination of dissolution kinetics over a range of temperature and pH.^49,50^ Sulfide minerals are also expected to be reactive in hydrothermal solutions, where dissolved H_2_S or HS^–^ could be formed through mineral dissolution and hydrolysis.^16^ Although mineral dissolution was not specifically examined in the present study, it is likely that the selected minerals could release dissolved metal ions and counterion species that potentially interact with organic molecules under hydrothermal conditions. However, the nonsubstantial effect observed from the metal salt-amine experiments seems to suggest that the mineral dissolution may not significantly influence the amine reaction rate and pathways under the studied experimental conditions.

The structural characteristics of the studied amines also contribute to their reactivity and stability. The aromatic ring in benzylamine can delocalize and form reactive carbocation intermediates, and the stabilization of benzyl cations can be enhanced by metal complexation.^51^ In contrast, nonaromatic structures such as cyclohexyl and cyclohexylmethyl are more resistant due to their stronger C–H/C–C bonds than those at the benzylic position. The combination of protonation, weak complexation, mineral surface protection, and electronic delocalization thus may provide a plausible molecular-level rationale for the observed persistence of amines. From a geochemical standpoint, these results imply that metal- and mineral-rich hydrothermal fluids can act as stabilizing rather than destructive environments for reduced organic nitrogen compounds. With proper temperature, pH, redox potential, and aqueous chemical composition, such systems may promote the long-term preservation and transport of amines within the deep crust and subseafloor. For example, deamination of amines could become less favorable in alkaline hydrothermal systems,^24,31^ which environment is widely recognized to be essential for the origin of life.^52^ These amine compounds could therefore accumulate and persist until they encounter suitable carbonyl or carboxylic partners, enabling reactions such as reductive amination with aldehydes or ketones, or condensation with carboxylic acids to form amides and related nitrogen-bearing biomolecules.^25,26,31,101^ Additionally, amines could have been useful in prebiotic synthesis of nitrogenous compounds (e.g., amino acids), acting as a source of ammonia rather than a constituent. Ammonia is known as a common starting material for producing amino acids (e.g., Strecker synthesis),^53^ and it has been observed as a major product from hydrothermal deamination of amines (e.g., benzylamine).^24^ The deamination reactivity of amines, particularly in the presence of minerals and metal salts, may also be an important characteristic for chemical evolution in early-Earth hydrothermal systems. Taken together, these findings reveal a broader geochemical view that natural hydrothermal systems not only provide a catalytic role in prebiotic synthesis but may also serve as a protective reservoir for biomolecular precursors in the subsurface environments.

## 4 Conclusions

This study reports that small aliphatic and aromatic amines remain largely stable under hydrothermal conditions in the presence of minerals and dissolved metal salts. Although minor shifts in conversion and product yields were observed, the overall reactivity remained largely unchanged, indicating that inorganic materials do not strongly influence the deamination of simple amines. The stability of amines in these metal- and mineral-rich fluids indicates that they could persist in subseafloor hydrothermal environments, enabling them to accumulate, circulate, and participate in chemical evolution. These results suggest that hydrothermal environments could have served not only as a dynamic medium for organic nitrogen transformation but also as a protective niche for biomolecule precursors during the chemical evolution on early Earth, and possibly, on other ocean worlds.

## Supplementary Material



## Notes

Product distribution of hydrothermal transformation of benzylamine in the absence and presence of minerals (Table S1); Product distribution of cyclohexylamine in the absence and presence of minerals (Table S2); Product distribution of cyclohexylmethylamine in the absence and presence of minerals (Table S3); Product distribution of benzylamine in the absence and presence of metal salts (Table S4); Product distribution of cyclohexylamine in the absence and presence of metal salts (Table S5); Product distribution of cyclohexylmethylamine in the absence and presence of metal salts (Table S6) (PDF)
