# Supplementary material for: Stability of Amines with Minerals and Metal Salts in Hydrothermal Fluids
Source: ACS Omega. 2026 Jul 20;11(30):45477–84. doi: 10.1021/acsomega.6c05119 (PMC13448923; doi:10.1021/acsomega.6c05119)
Supplement: Supplementary file 1 [file ao6c05119_si_001.pdf]

Supporting Information

Stability of Amines with Minerals and Metal Salts in Hydrothermal Fluids

Yiju Liao[1], Alexandria Aspin[1], and Ziming Yang[1*]

[1]Department of Chemistry, Oakland University, Rochester, MI 48309, USA.

Corresponding author: Ziming Yang (zimingyang@oakland.edu)

Table S1. Major product distribution (mole%) of hydrothermal transformation of benzylamine in the absence and presence of minerals at 250 ºC after 24 h. Errors represent one standard deviation of replicate experiments.

	**Benzylamine (mole%)**	**Benzyl alcohol (mole%)**	**Dibenzylamine (mole%)**	**Benzaldehyde (mole%)**
**No mineral**	68.3±6.4	10.0±3.7	20.5±4.7	1.2±0.2
**Al_2O_3**	71.2±7.2	8.4±4.8	19.1±5.6	1.3±0.3
**Fe_2O_3**	74.1±1.2	4.6±1.0	20.3±1.1	1.0±0.1
**$Al(OH)_3$**	65.1±3.9	7.3±3.5	26.3±6.5	1.2±0.2
**Fe_3O_4**	66.7±7.2	5.6±1.5	26.4±5.4	1.3±0.1
**FeS**	64.3±6.5	5.9±2.4	28.7±7.9	1.1±0.1
**$Mg(OH)_2$**	63.4±6.5	9.2±5.2	25.9±3.4	1.5±0.4
**ZnS**	74.1±8.7	5.6±0.9	19.3±3.5	1.0±0.2

Table S2. Major product distribution (mole%) of hydrothermal transformation of cyclohexylamine in the absence and presence of minerals at 250 ºC after 72 h. Errors represent one standard deviation of replicate experiments.

	**Cyclohexylamine (mole%)**	**Cyclohexanol (mole%)**	**Dicyclohexylamine (mole%)**	**Cyclohexene (mole%)**
**No mineral**	73.8±3.2	9.1±2.2	1.6±0.1	5.7±0.8
**Al_2O_3**	72.7±1.7	7.5±1.1	1.7±0.1	7.4±1.3
**Fe_2O_3**	73.4±2.0	7.9±1.5	0.9±0.1	6.5±1.1
**$Al(OH)_3$**	69.0±4.5	10.3±2.6	2.6±0.2	5.9±0.7
**Fe_3O_4**	72.0±3.2	6.5±0.4	1.1±0.1	4.9±0.3
**FeS**	70.2±2.9	10.0±1.5	2.3±0.1	5.6±0.9
**$Mg(OH)_2$**	68.1±5.2	10.2±2.4	2.4±0.3	6.6±0.4
**ZnS**	70.1±4.4	8.8±0.7	1.4±0.2	4.8±1.2

Table S3. Major product distribution (mole%) of hydrothermal transformation of cyclohexylmethylamine in the absence and presence of minerals at 250 ºC after 72 h. Errors represent one standard deviation of replicate experiments.

	**Cyclohexylmethylamine (mole%)**	**Cyclohexylmethanol (mole%)**	**Dicyclohexylmethylamine (mole%)**
**No mineral**	54.5±3.2	13.8±0.9	12.9±0.8
**Al_2O_3**	59.2±5.4	12.8±1.2	14.3±1.1
**Fe_2O_3**	53.9±4.2	11.0±0.5	13.4±0.6
**$Al(OH)_3$**	48.5±4.8	12.0±0.8	14.6±0.3
**Fe_3O_4**	52.4±1.6	10.4±0.3	13.6±0.7
**FeS**	54.1±3.1	8.2±0.4	9.5±2.1
**$Mg(OH)_2$**	55.0±2.5	9.6±0.4	15.0±0.4
**ZnS**	46.7±6.9	11.4±0.6	16.4±1.5

Table S4. Major product distribution (mole%) of hydrothermal transformation of benzylamine in the absence and presence of metal salts at 250 ºC after 4 h. Errors represent one standard deviation of replicate experiments.

	**Benzylamine (mole%)**	**Benzyl alcohol (mole%)**	**Dibenzylamine (mole%)**	**Benzaldehyde (mole%)**
**No salts**	84.4±8.7	3.5±0.5	11.3±1.9	0.9±0.2
**$AlCl_3$**	76.4±3.8	10.4±3.5	12.2±0.2	1.0±2.3
**$FeCl_3$**	78.1±3.7	8.3±2.7	13.1±1.5	0.5±2.3
**$CuCl_2$**	70.7±7.1	7.5±1.4	17.5±3.4	4.3±1.8
**$MgCl_2$**	76.4±1.6	12.2±4.5	10.5±0.4	0.9±3.4
**$ZnCl_2$**	75.1±1.3	12.4±5.0	11.4±1.0	1.1±5.3
**NaCl**	83.6±3.1	5.0±0.7	10.4±0.2	1.0±2.6

Table S5. Major product distribution (mole%) of hydrothermal transformation of cyclohexylamine in the absence and presence of metal salts at 250 ºC after 24 h. Errors represent one standard deviation of replicate experiments.

	**Cyclohexylamine (mole%)**	**Cyclohexanol (mole%)**	**Dicyclohexylamine (mole%)**	**Cyclohexene (mole%)**
**No salts**	87.1±5.3	8.3±1.1	0.5±0.1	4.4±0.9
**$AlCl_3$**	86.2±3.2	7.1±0.8	0.6±0.1	5.4±0.7
**$FeCl_3$**	86.3±7.1	6.9±1.3	0.7±0.1	5.5±0.3
**$CuCl_2$**	85.8±4.3	10.9±2.5	0.8±0.1	3.8±0.8
**$MgCl_2$**	86.2±2.7	7.3±0.7	0.6±0.0	5.4±0.3
**$ZnCl_2$**	84.3±5.4	7.7±0.4	0.7±0.1	6.6±0.4
**NaCl**	86.1±5.6	7.6±0.8	0.6±0.1	5.2±0.2

Table S6. Major product distribution (mole%) of hydrothermal transformation of cyclohexylmethylamine in the absence and presence of metal salts at 250 ºC after 24 h. Errors represent one standard deviation of replicate experiments.

	**Cyclohexylmethylamine (mole%)**	**Cyclohexylmethanol (mole%)**	**Dicyclohexylmethylamine (mole%)**
**No salts**	80.6±4.5	8.7±1.1	5.7±0.3
**$AlCl_3$**	83.9±3.2	7.1±0.8	4.0±0.2
**$FeCl_3$**	79.6±6.8	8.1±0.5	5.4±0.2
**$CuCl_2$**	77.9±7.7	10.8±0.4	7.8±1.1
**$MgCl_2$**	83.5±2.5	9.1±0.6	7.6±0.9
**$ZnCl_2$**	80.5±5.4	11.2±0.9	4.6±0.3
**NaCl**	81.6±1.9	8.9±0.3	5.0±0.4
